# Professionals' treatment goals for long-term benzodiazepine and Z-drugs management: a qualitative study

**DOI:** 10.3399/BJGPO.2023.0034

**Published:** 2024-02-07

**Authors:** Pauline Van Ngoc, Melissa Ceuterick, Jean-Luc Belche, Beatrice Scholtes

**Affiliations:** 1 Research Unit of Primary Care and Health, Department of General Medicine, University of Liège, Liège, Belgium; 2 Hedera, Department of Sociology, Ghent University, Ghent, Belgium

**Keywords:** benzodiazepines, primary health care, goal-oriented care, qualitative research

## Abstract

**Background:**

Benzodiazepines and Z-drugs (BZD/Z) are frequently prescribed for longer than recommended. Through their interactions with patients taking BZD/Z, primary care and mental health professionals play a key role in the management of this medication.

**Aim:**

To explore how primary care and mental health care professionals set treatment goals with users of long-term BZD/Z.

**Design & setting:**

A qualitative study using semi-structured interviews with professionals from mental health, addiction care, and primary care practices in Belgium.

**Method:**

Semi-structured interviews were conducted, online and in person, with 24 professionals working in mental health and primary care. Inductive thematic content analysis was performed.

**Results:**

Seven themes were identified from the analysis. Professionals tended not to use the Diagnostic and Statistical Manual of Mental Disorders, Fifth Edition (DSM-V) standard to diagnose a BZD/Z substance use disorder. They described criteria based on their experience. They identified diverse types of patients that influence their choice of treatment goals. Professionals appeared to position themselves according to their own treatment goals for their patients, either by promoting the goal of abstinence or harm reduction. Some of them reported feeling trapped into continuing to prescribe and considered BZD/Z withdrawal to be difficult. Some were afraid to engage in a conversation that might break the bond of trust with the patient. Few professionals mentioned patient participation in the treatment goal setting. They asked for targeted withdrawal recommendations, perceiving the current recommendations to be too broad.

**Conclusion:**

Whether primary care or mental health care professionals are more in favour of a total abstinence or a harm reduction approach to BZD/Z, professionals should be guided towards greater patient participation in setting and evaluating goals with patients taking BZD/Z.

## How this fits in

Benzodiazepines and Z-drugs (BZD/Z) are widely prescribed and for longer periods than currently recommended (2–4 weeks). In Belgium, a gap between guidelines and clinical practice persists.^1^ This qualitative study explored how primary care and mental health care professionals set treatment goals with users of long-term BZD/Z through the lens of goal-oriented care.

## Introduction

Benzodiazepines and Z-drugs (BZD/Z) are broadly prescribed, and inappropriate consumption constitutes a major public health concern.^2,3^ Belgian guidelines recommend prescribing BZD/Z as a last resort at the lowest possible dose for a period of 1–4 weeks.^4,5^ Nevertheless, in 2018, the results of the National Health Interview Survey showed that 14.9% of participants aged ≥65 years had taken a BZD/Z in the 24 hours preceding the survey.^6^ Long-term usage of benzodiazepines (>6 months)^7^ can lead to adverse side effects such as psychological, cognitive, and physical effects including vertigo, ataxia leading to falls, and dysarthria.^8^ Furthermore, physiological and psychological dependence can be induced,^3,8^ which can lead to misuse, abuse, or to a substance use disorder (SUD), as described in the Diagnostic and Statistical Manual of Mental Disorders, Fifth Edition (DSM-V).^9^ Likewise, Z-drug use is also associated with adverse effects such as delirium, dementia, fractures, or road traffic accidents.^10–12^ Even short-term use can engender side effects, including impairment in psychomotor and cognitive functioning, and can lead to tolerance.^13^

In 2020, a Belgian report showed that guidelines are not being followed appropriately, especially in terms of prescription duration.^14^ These gaps between guidelines and practice illustrate the difficulties professionals face regarding long-term BZD prescriptions and deprescribing.^15^ An alternative approach to address these difficulties could be to prioritise patient goals.

While biomedicine is founded on the disease–outcome paradigm, where each disease is treated separately following guidelines and population goals, the goal-oriented care (GOC) approach is part of a dynamic process of collaboration and co-creation between the patient and the professional based on the patient’s needs, preferences, and values.^16,17^ This approach can be divided into three steps during a consultation.^17^ The first is goal elicitation where professionals and patients work on understanding each other’s expectations. Then goal setting is defined based on the patient’s needs and preferences. This is followed by goal evaluation, which requires working on measurable common goals.^17^ Both parties must be prepared to change their paradigm of care; this may require professional training on how to define the objectives of care with their patients^18^ and on how to help them share their needs and preferences.^16^ This approach has been predominantly employed and studied among patients with chronic conditions.^17^

Given the challenging nature of BZD/Z withdrawal and the high likelihood that these types of patients already have complex physical and mental health needs,^19,20^ a GOC approach seems appropriate. Nevertheless, as described above, the process is not necessarily easy and greater understanding of the current state of affairs would be helpful. We therefore posed the research question: how do primary care and mental healthcare professionals in Belgium currently set treatment goals with users of long-term BZD/Z?

## Method

A semi-structured interview study was conducted among healthcare professionals using an interview guide (Supplementary Box S1) based on the model of healthcare accessibility by Andersen.^21^ The guide was developed by three researchers (MC, BS, PV) and was piloted with two interviews, one in each language.

Participants were recruited via Belgian mental health care and primary care networks and via a Belgian medical professional newspaper. Efforts were made to achieve a diverse sample in terms of geographical regions, sex, type of profession, and practice (Table 1). We aimed to recruit at least two participants per profession (GP, nurse, social worker, psychiatrist, psychologist). Only professionals who were interested in participating contacted the research team; therefore, we have no information on those who refused to participate. One interview per participant was held, one-to-one, at the participant’s workplace or by video conference. The interviews took place between July 2021 and January 2022, and the average duration of the interviews was 68 minutes.

**Table 1.** Participant characteristics

Code	Date	Sex	Professional background	Type of practice	Location	Region	Interviewer	Online or in person
RESP1	14-07-21	Female	GP	Addiction care	Urban	Flanders	MC	Online
RESP2	20-09-21	Male	GP	Addiction care	Urban	Flanders	MC	In person
RESP3	13-10-21	Male	Social worker	Addiction care	Urban	Flanders	AV	In person
RESP4	20-10-21	Male	GP	Addiction care	Urban	Flanders	MC	In person
RESP5	22-10-21	Male	Psychiatrist	Addiction care	Urban	Flanders	MC	In person
RESP6	03-11-21	Female	GP	Addiction care	Urban	Flanders	AV	In person
RESP7	04-11-21	Female	GP	Addiction care	Urban	Flanders	MC	In person
RESP8	17-11-21	Female	GP	Primary care	Urban	Flanders	MC	Online
RESP9	30-11-21	Female	Psychiatrist	Addiction care	Urban	Flanders	MC	Online
RESP10	09-12-21	Female	GP	Primary care	Urban/rural	Flanders	MC	Online
RESP11	29-12-21	Female	Nurse	Mental health care	Urban	Flanders	AV	In person
RESP12	07-01-22	Male	Psychologist	Mental health care	Urban	Flanders	AV	In person
RESP13	07-01-22	Male	Psychiatrist	Mental health care	Urban	Flanders	AV	In person
RESP14	08-07-21	Female	GP	Primary care	Rural	Wallonia	PV	In person
RESP15	05-08-21	Male	GP	Primary care	Urban	Wallonia	PV	In person
RESP16	23-08-21	Female	Psychologist	Addiction care	Urban	Brussels	PV	In person
RESP17	23-08-21	Female	GP	Addiction care	Urban	Brussels	PV	In person
RESP18	06-09-21	Female	Social worker	Addiction care	Rural	Wallonia	PV	In person
RESP19	09-09-21	Female	Nurse	Addiction care	Urban	Brussels	PV	In person
RESP20	10-09-21	Male	Psychiatrist	Mental health care	Rural	Wallonia	PV	In person
RESP21	13-09-21	Male	GP	Primary care	Urban	Brussels	PV	In person
RESP22	21-09-21	Male	Psychologist	Addiction care	Rural	Wallonia	PV	In person
RESP23	22-09-21	Male	Psychiatrist	Addiction care	Urban	Brussels	PV	In person
RESP24	19-11-21	Female	Social worker	Addiction care	Rural	Wallonia	PV	Online

Date format = DD-MM-YY.

Interviews were conducted by the first author (PV, female, PhD student and psychologist by training, *n* = 11), the second author (MC, female, postdoc researcher and medical anthropologist, *n* = 8), and a research team member (AV, retired GP, female, *n* = 5). All three had been trained in interviewing.

Participants signed a consent form before the interview. All the interviews were audio-recorded and transcribed, and identifying information was removed. Transcripts were proofread for accuracy; they were not returned to participants for comments or correction. Field notes were taken but did not add anything new to the dataset.

Team meetings were held every fortnight to discuss progression of data collection.

### Data analysis

Inductive thematic content analysis was performed using NVivo (version 12). A first code matrix with recurring themes was produced and was discussed by the core research team (PV, MC, BS). The Dutch-speaking researcher (MC) coded the interviews in Dutch and the French-speaking researcher (PV) coded the interviews in French. To ensure that the coding was similar, two interviews in each language were coded by both coders using the same coding tree (Supplementary Table S1). Several discussions were organised between the coders to review the themes in an iterative process. Data saturation was attained when all the data had been analysed.^22^

## Results

A purposive sample of 24 participants from the three regions of Belgium (Brussels, Flanders, and Wallonia) was recruited from different professional backgrounds (Table 1). In total, 11 interviews were carried out in French and 13 in Dutch (Table 1) with GPs (*n* = 11), psychologists (*n* = 3), psychiatrists (*n* = 5), nurses (*n* = 2), and social workers (*n* = 3).

The following seven themes emerged from the data about the treatment goals:

- Diagnosis and recognition of a BZD/Z SUD;
- Position concerning their treatment goals;
- Goal of abstinence;
- Harm reduction;
- Feeling trapped;
- Patient participation;
- Targeted recommendations.

### Diagnostic and recognition of a BZD/Z SUD

Participants did not always use criteria, such as those described in the DSM-V,^9^ to diagnose a BZD/Z SUD. The participants spoke rather of a ‘feeling’ and identified a change in the patient’s behaviour to obtain more prescriptions, a non-respect of the prescribed dose, and where the BZD/Z becomes necessary for their proper functioning.

‘*There are behaviours that are adopted by the patient to get the prescription and I have to say also that I don’t know if this will be true for all physicians, but I also feel when there is something wrong.*’ (RESP14, GP, primary care, rural)

‘*We really speak of extreme use when people regularly go medical shopping, when they do not actually follow the prescribed doses at all or they take them at the wrong times, things like that.*’ (RESP12, psychologist, mental health care, urban)

The participants explained how they differentiate between the different types of patients, for example, an older person who takes a BZD/Z every evening or a person who takes BZD/Z in addition to other drugs to become ‘high’. This classification influenced the choice of professionals in the patient’s treatment goals. Indeed, the older person who takes their BZD every night will be less likely to be associated with a SUD than a person who takes it for recreational purposes:

*‘I’d say, if someone of eighty says, “I take half of a Stilnoct* [zolpidem] *every day.” And I feel good about that.*’ (RESP1, GP, addiction care, urban)

*‘For people who are completely slowed down. There, we see that the dosage is not adequate or that there is an abuse. Because they tell us that too … “I took some pills last night, I did that, and I don’t remember anything.”’* (RESP19, nurse, addiction care, urban)

### Position concerning their treatment goals

Most of the participants positioned themselves, and their professional practices, within their own global treatment goals. Some professionals working in primary care or outpatient services focused on harm reduction by continuing to prescribe a low or high dose of BZD/Z to maintain the relationship with patients who needed specialised support in terms of mental health, addiction, or social inclusion. On the other hand, others were in favour of abstinence, such as professionals working in residential care where the patient is not allowed to take any substance before or during their stay in the facility.

*‘And between the two centres* [one advocating total abstinence versus one advocating harm reduction], *there was sometimes a bit of a cold war … Today, there is a situation of coexistence between those different visions.’ (*RESP1, GP, addiction care, urban)

Professionals promoting complete abstinence and those supporting harm reduction strategies seemed somewhat distinct. One participant mentioned a dogmatic vision for those who believed in abstinence and a ‘*cold war*’ between these approaches. There seemed to be a coexistence between these distinct beliefs, or for some even ‘professional identities’ concerning what was possible for patients who have been taking BZD/Z for long time.

‘*I don’t know if we can really believe in that “harm reduction”.’* (RESP10, GP, primary care, urban/rural)

### Goal of abstinence

Some of the participants who described a belief in abstinence as an outcome work in institutions that have the same approach and patients know that if they are admitted, they will have to be abstinent at admission or become abstinent:

*‘So that is also a very clear, very clear message that people know: if you are admitted, it is for total abstinence.’* (RESP5, psychiatrist, addiction care, urban)

They considered a patient to have recovered when they are happy to be without any substance and have established other connections:

*‘Someone’s recovery, for us who have a goal of abstinence and reintegration, is someone who is happy to live soberly and has managed to make other connections elsewhere and becomes satisfying for themselves and others.’* (RESP24, social worker, addiction care, rural)

Within this context, a participant mentioned the value they perceived for the patient of experiencing life without any substances:

*‘The reason for this* [centre’s policy of demanding abstinence before admission] *is that, in carrying out daily tasks sober, i.e. without products, people will rediscover themselves.* […] *So, we’re going to work with that, give ourselves room for error, where does that come from? How can you do differently? It’s a sentence that comes up every day. How can you do otherwise?’* (RESP18, social worker, addiction care, rural)

### Harm reduction

For participants in favour of harm reduction, they mentioned the intention that their patients achieve a degree of stability in their BZD/Z consumption. Some explained their experience of patients taking high doses of benzodiazepines with high tolerance. Some of them did not consider taking BZD/Z for a prolonged period to be a problem.

They described that the aim was for their patients to establish stability in their use and gain a quality of life to maintain ‘*a good addiction, or the least bad’* (RESP21, GP, primary care, urban).

*‘This* [a person who has established a stable use of BZD] *is someone who is not going to put themselves at risk financially, legally, judicially, professionally, familially with their use.’* (RESP15, GP, primary care, urban)

Some participants described the importance for them to maintain the therapeutic relationship with the patient and that they welcomed patients unconditionally, whatever their consumption or their condition. They considered this to be more valuable than following the recommendations on BZD/Z prescriptions:

*‘Keeping the link long enough to possibly stabilise the patients and above all to allow them to get back on track for those who are socially disengaged.’* (RESP17, GP, addiction care, urban)

Furthermore, if they were reducing BZD doses, they described wanting to accommodate the patient’s pace, which can be slower than the withdrawal guidelines:

*‘I agreed to become less true to my principles as a prescriber, realising the advantage of keeping a link.’* (RESP17, GP, addiction care, urban)

*‘That you have been able to achieve some kind of damage limitation in their use, and that is often a mix of uppers and downers.’* (RESP2, GP, addiction care, urban)

### Feeling trapped

Some participants reported feeling stuck with BZD/Z deprescription. They observed that sometimes even small dose reductions can be very complex and that it is difficult to apply the guidelines in a universal way. Moreover, they reported that withdrawal is, in itself, very difficult for the patient:

*‘Benzodiazepine withdrawal is a dreadful mess. We’re stuck.’* (RESP21, GP, primary care, urban)

*‘A small decrease often takes an implausible turn.’* (RESP23, psychiatrist, addiction care, urban)

Some participants described difficulties talking with their patients about their long-term BZD/Z use owing to fear of breaking the trusting relationship with their patients, or of upsetting the status quo established with the drug (particularly for older patients) as well as the fear of not being able to offer an alternative solution.

*‘Because, as I said earlier, there are patients with whom we get on very well and with whom the relationship is very good. And they are sometimes the worst ones to tell because there is a real risk of a breach of trust … And also, alongside the fear of a breach of trust, there’s also the fear of not being able to offer anything else. And the fear of breaking a balance that is present. The elderly person who has been taking zolpidem for 10 years and then there you go …*’ (RESP14, GP, primary care, rural)

### Patient participation

Participants explained how they take time with their patients to accompany them in their journey. They aimed to find out where they are starting from and where they want to go to. But it was not clear whether the patient was involved in this decision-making process. For some, they expected a request, an active demand from the patient to decrease the doses:

*‘We accompany them wherever they want to go.’* (RESP16, psychologist, addiction care, urban)

*'You need to know where they start from and where they want to go.’* (RESP21, GP, primary care, urban)

One psychiatrist stated that they asked patients what they thought about their treatment goals:

*‘Which ones do I set? I always ask them what they think.’* (RESP13, Psychiatrist, mental health care, urban)

### Targeted recommendations

Concerning the withdrawal, some participants described their difficulties applying guidelines that they considered were not appropriate for their patient group. Some changed the molecules to the equivalent of diazepam and decreased more slowly than the recommendations, depending on the patient’s profile. In addition, some prescribers mentioned the need to keep the BZD/Z for a long time, without being able to stop it:

*‘It’s a dependence, no more than a maximum of six diazepam equivalent per day. And then, we will really decrease, but depending on the duration of the addiction, depending on the patient’s profile, his real motivation, etc. sometimes it will be very slow.’* (RESP17, GP, addiction care, urban)

A participant suggested making targeted recommendations for patients who did not fit into the current guidelines.

*‘Good practices are realistically made to measure for a group of benzo-dependent people who do not fit into the current frameworks.’* (RESP2, GP, addiction care, urban)

## Discussion

### Summary

Our results show that patients are diagnosed according to professional criteria, not necessarily recognised guidelines. Professionals seem to have opposing views on treatment goals, some favouring abstinence and others harm reduction. The patient appears to be hardly involved in the choice of the treatment goal and the process seems to be more professional-centred. Indeed, it is the professional who felt trapped between what the patient is able to do and what the guidelines recommend. This qualitative study explored how primary care and mental health professionals set their treatment goals with users of long-term BZD/Z.

### Strengths and limitations

This study is part of a larger project, BENZOCARE, focusing on the experience of professionals and patients regarding the use of BZD/Z and the accessibility of care. The results presented here were collected in the context of that study, which in its essence was not focused exclusively on GOC. The interview guide was therefore somewhat broad and these results represent a part of the data collected. If we had created an interview guide tailored to GOC, we may have had different results but there may also have been a tendency towards social desirability bias given the push towards GOC in Belgium at the moment. We feel, therefore, that our results reflect the way BZD/Z are managed in primary care and mental health services.

The study was conducted in Belgium and data were collected in French and in Dutch (sometimes in local dialect). In order to facilitate comparability and discussion within the team and develop a global understanding of the data, the Dutch transcripts were translated into French after having been analysed separately by two coders in the original language. The team met several times during data collection and analysis to develop a coding tree and to achieve a shared deep understanding of the interview transcripts. Nevertheless, some misinterpretations may remain. We aimed to achieve a heterogeneous sample. This gave us a richness in terms of experiences but somewhat limits the representativeness of each professional profile included. This study included a possible selection bias in the willingness of professionals to answer questions about BZD/Z and a social desirability bias in the answers they gave.

### Comparison with existing literature

There seems to be two broad points of view: being in favour of total abstinence with zero tolerance; or an approach prioritising harm reduction. This dichotomy has also been observed in mental health care literature in opioid treatment, where there are either abstinence programmes or harm reduction programmes.^23^ Whereas the total abstinence approach to addiction treatment prevailed, harm reduction has made it possible to offer a choice of treatment goals.^24^ Despite the existence of these different approaches, a study conducted in the context of treatment for alcoholism showed that some patients internalise total abstinence as the only goal and therefore see alternative options as a second choice or a failure.^19^ Faced with this diversity of approaches, Gallagher and colleagues proposed a paradigm shift for professionals to view treatment and recovery differently.^23^ The authors conceptualised recovery on a spectrum that is determined by patients and that is not a projection of professional or institutional values.

In our results, patients' expectations appeared not to be the starting point for setting treatment goals. Some professionals appeared to find it difficult to start a conversation about BZD/Z. Previous research has demonstrated that prescriber behaviour is influenced by assumptions concerning patients’ expectations, motivation, and adaptability, such as anticipating patient resistance to initiating a deprescribing process.^20,25^ This has also been described for insomnia management where they recommend that practitioners elucidate patients’ beliefs and expectations.^26^ For this purpose, a dialogue between the prescriber and the patient is necessary.^27^ This tendency to avoid engaging in dialogue seemed to be present in our sample as well and is also experienced on the other side of the desk, with some patients feeling stuck or imprisoned during their withdrawal from BZD/Z.^15,28^ Patients are unaware of the potential problems associated with their BZD/Z use and do not realise that they are addicted to these drugs, until the day they try to stop.^15^

Furthermore, in our study, some prescribing participants mentioned their fear of breaking the trusting relationship if they initiated the conversation about BZD/Z. This is consistent with other studies about users of BZDs, where physicians were reported to have experienced the same discomforts.^20,29^ While communication between doctor and patient is positively correlated with patient adherence to treatment,^30^ prescribers seem to be struggling to know how to engage the talk about BZD/Z.^27^ They declared having insufficient time, and need training for communication and negotiation skills to discuss BZD.^31^ The challenges professionals face communicating with patients regarding this problem is understandable and models have been developed to structure such discussions and help develop goals together. The ‘three-talk model’ proposes three stages in the consultation: first, ‘team talk’ where the professional and the patient work together to describe the possible choices, offer support, and describe the objectives; second, ‘option talk’ where alternatives are discussed, and third ‘decision talk’ where decisions are made based on patient preferences.^32^

As some participants mentioned, tapering off BZD/Z can be very difficult. However, one of the benefits of withdrawal mentioned by professionals is the opportunity for the patient to get to know themselves without substances and to make new connections. This can be contrasted with a study on the psychotropic self that described how both psychiatrists and patients want to make the patient feel ‘normal’ and achieve a ‘normal’ patient self, except that the definition of ‘feeling normal’ differs considerably between doctor and patient.^33^ Patients have built their ‘self’ in the context of addictions and want to continue to be that ‘self’. However, with the treatment they receive, they are led to a different ‘self’. The authors question what a successful treatment is, knowing that the treatment goals differ between patients and professionals.^33^ This underlines the need for the two parties to move together in the same direction. This would involve open dialogue with the patient and a GOC approach concerning whether to keep the same dose, stabilise it, or reduce it.

In general, our results point to a lack of patient-centred care in the management of BZD/Z SUD. Indeed, in a systematic review in 2018, the authors did not find any studies that included a BZD-tapering intervention that was defined as specifically using the concept of patient-centred care. Nevertheless, among their results 20 interventions were based on patient-centred dimensions of the approach, focusing on patient information, professional–patient communication, and provider characteristics.^34^ Among them, a study of an educational intervention had a positive impact on shared decision-making for benzodiazepine overuse in older people.^35^

Today, primary care literature is increasingly focusing on the GOC approach; however, less focus is afforded in the literature to GOC and BZD/Z use. Similarly, GOC or elements of it, were rarely mentioned by our participants. GOC requires working in partnership with users, families, healthcare professionals, the public, and other service providers.^36^ The approach can have a positive impact on the patient experience, the wellbeing of professionals, lead to a reduction in healthcare costs, and improve population health.^17^ In light of our results and the burden of BZD/Z prescribing and deprescribing, it appears important to explore and study the GOC approach in the context of both initial prescriptions of BZD/Z and deprescription.

### Implications for research and practice

Total abstinence appeared to be complex to implement universally given the diversity of patients taking BZD/Z, especially regarding complete withdrawal within a few weeks. Some professionals asked for recommendations for specific populations for BZD/Z tapering off. Implementing the GOC approach in the BZD/Z guidelines by taking into consideration the challenges faced by clinicians and integrating needs, preferences, and values of patients could be considered in future studies and in practical guidance.

Professionals reported feeling trapped by the issue of BZD/Z, which is detrimental to both them and their patients. A GOC approach could help align treatment and support with the patient’s needs and desires and simultaneously support professionals navigating this complex issue.

To our knowledge, while the literature on GOC in primary care is growing, this article is one of the first to study the experiences of professionals concerning BZD/Z use through the prism of the GOC approach.

Our results have concluded that the DSM-V does not appear to be used by professionals to diagnose a BZD/Z SUD and instead they use their instincts. Participants clearly positioned themselves between an abstinence or harm reduction goal. They described feeling trapped in the prescription of BZD/Z and patient involvement in decision making is not clear. Participants requested recommendations that were specific to certain patient groups. We feel that by recognising or diagnosing a BZD/Z SUD using criteria already defined in the DSM-V could increase transparency between the patient and professional. Co-creating goals according to the patient’s needs, preferences, and values could help navigate the challenges professionals feel concerning harm reduction or total abstinence making them feel less trapped.

Renewed emphasis in professional training on the patient experience of BZD/Z could be undertaken to promote patient empowerment in the context of BZD/Z prescription and deprescription. Ongoing training in the GOC approach and in communication techniques for healthcare professionals is also important. Future studies should focus more on the patient’s lived experience of BZD/Z dependence and withdrawal and the relationship with professionals.

## Data

The dataset relied on in this article is available from the corresponding author on reasonable request.
